# Bacterial glycosylation, it’s complicated

**DOI:** 10.3389/fmolb.2022.1015771

**Published:** 2022-09-30

**Authors:** Christine M. Szymanski

**Affiliations:** Department of Microbiology and Complex Carbohydrate Research Center, University of Georgia, Athens, GA, United States

**Keywords:** glycoconjugates, carbohydrates, polysaccharides, *Campylobacter jejuni*, phase-variation

## Abstract

Each microbe has the ability to produce a wide variety of sugar structures that includes some combination of glycolipids, glycoproteins, exopolysaccharides and oligosaccharides. For example, bacteria may synthesize lipooligosaccharides or lipopolysaccharides, teichoic and lipoteichoic acids, N- and O-linked glycoproteins, capsular polysaccharides, exopolysaccharides, poly-N-acetylglycosamine polymers, peptidoglycans, osmoregulated periplasmic glucans, trehalose or glycogen, just to name a few of the more broadly distributed carbohydrates that have been studied. The composition of many of these glycans are typically dissimilar from those described in eukaryotes, both in the seemingly endless repertoire of sugars that microbes are capable of synthesizing, and in the unique modifications that are attached to the carbohydrate residues. Furthermore, strain-to-strain differences in the carbohydrate building blocks used to create these glycoconjugates are the norm, and many strains possess additional mechanisms for turning on and off transferases that add specific monosaccharides and/or modifications, exponentially contributing to the structural heterogeneity observed by a single isolate, and preventing any structural generalization at the species level. In the past, a greater proportion of research effort was directed toward characterizing human pathogens rather than commensals or environmental isolates, and historically, the focus was on microbes that were simple to grow in large quantities and straightforward to genetically manipulate. These studies have revealed the complexity that exists among individual strains and have formed a foundation to better understand how other microbes, hosts and environments further transform the glycan composition of a single isolate. These studies also motivate researchers to further explore microbial glycan diversity, particularly as more sensitive analytical instruments and methods are developed to examine microbial populations *in situ* rather than in large scale from an enriched nutrient flask. This review emphasizes many of these points using the common foodborne pathogen *Campylobacter jejuni* as the model microbe.

## Introduction

**FIGURE 1:**
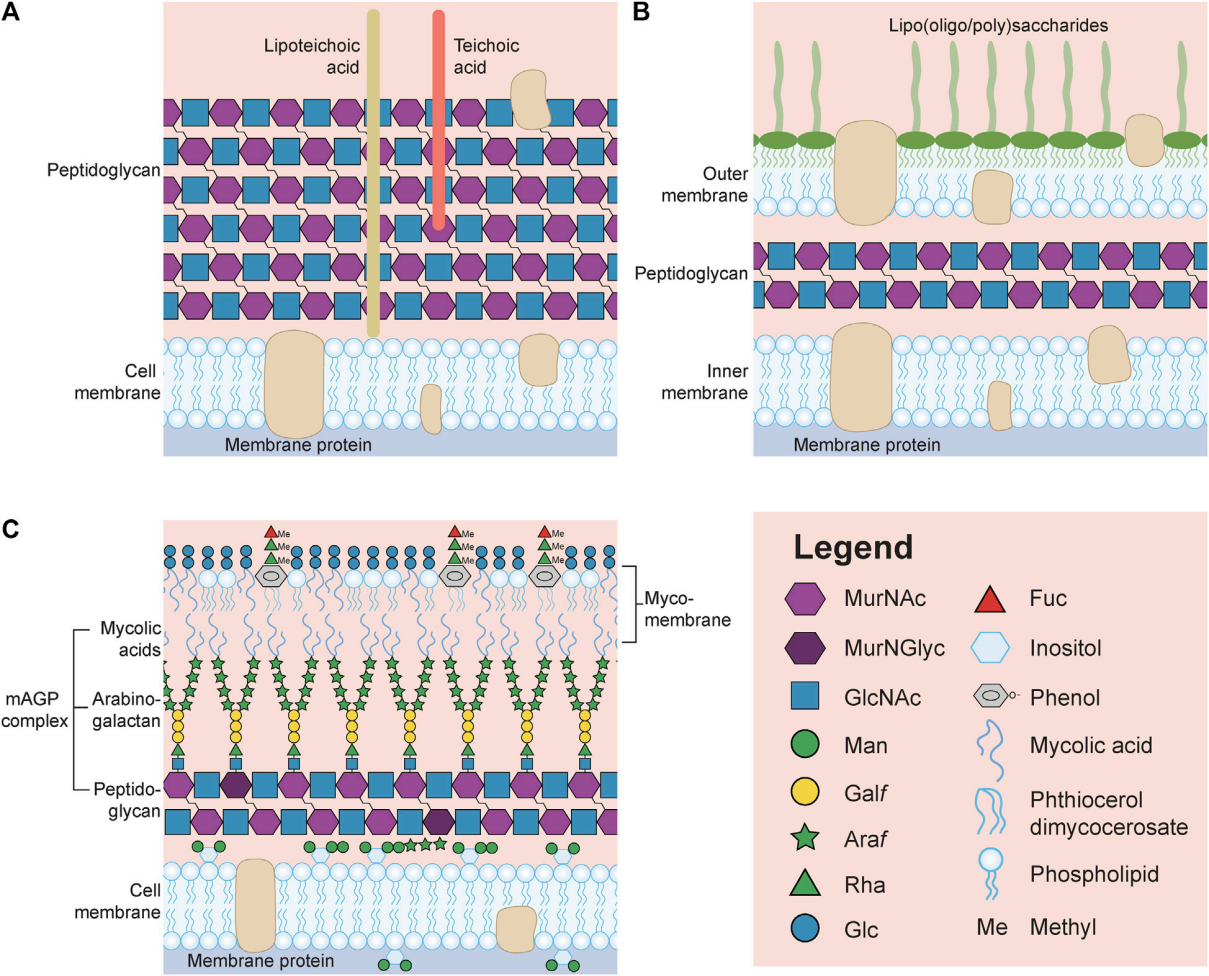
General representation of the surface of a Gram-positive **(A)** and Gram-negative **(B)** bacterium. **(C)** Schematic of the surface of *Mycobacterium tuberculosis* unable to be identified by the Gram stain. Note that many structures, including capsular polysaccharides and glycoproteins, have been omitted in order to reduce the complexity of the image. Also, lipoteichoic acids, teichoic acids and lipooligosaccharides/lipopolysaccharides are not drawn with the symbol nomenclature for glycans (SNFG, https://www.ncbi.nlm.nih.gov/glycans/snfg.html) due to the limitless structures that could be drawn for each glycoconjugate. MurNAc, N-acetylmuramic acid; MurNGlyc, N-glycolylmuramic acid; GlcNAc, N-acetylglucosamine; Man, mannose; Galf, galactofuranose; Araf, arabinofuranose; Rha, rhamnose; Glc, glucose; Fuc, fucose.

No Microbiology textbook or review would be complete without becoming familiar with the multitude of carbohydrate structures bacteria are capable of synthesizing, and in many cases are unable to survive without (for further reading, please see (Whitfield et al., 2022)). To begin, the peptidoglycan is an essential structure synthesized by all microbes and is necessary to both maintain cell shape and prevent cell lysis due to turgor pressure. Peptidoglycans are comprised of linear chains of alternating N-acetylglucosamine (GlcNAc) and N-acetylmuramic acid (MurNAc) that are held together by short peptide cross-links (Figure 1). These sugars can be further modified by O-acetyl groups to protect against lysozyme, a muramidase of the innate immune system that cleaves the β-(1→4) linkages between GlcNAc and MurNAc; and the structure is recognized by microbe-associated molecular pattern (MAMP) Toll-like receptor 2 (TLR2). Peptidoglycans are also the target of the first antibiotic that was identified by Sir Alexander Fleming in 1928 from the *Penicillium* fungus (Henderson, 1997). Since then, other beta-lactams and their derivatives, such as carbapenems and cephalosporins, as well as β-lactamase inhibitors, have been used to save millions of lives from fatal bacterial infections. The peptidoglycan structures are also targets of lysins for bacteriophage escape after the action of holins which create holes in the membrane for lysin release.

Beyond the ubiquitous peptidoglycan, other bacterial glycans are either unique to gram-positive (e.g., lipoteichoic acids and teichoic acids, Figure 1A) or Gram-negative (e.g., lipooligosaccharides or lipopolysaccharides, Figure 1B) microbes, or can be associated occasionally with both (e.g., capsules, O-linked glycoproteins). Alternatively, mycobacteria do not fit into either designation, and this is not surprising if one simply looks at the unique architecture of their cell wall (*M. tuberculosis* is provided as an example in Figure 1C). The classification of bacteria, developed by Hans Christian Gram is based on the observation that Gram-positive microbes retain the Gram stain (crystal violet) within their thick surface peptidoglycan layer, while the stain is readily rinsed away due to the presence of a second membrane covering the thin peptidoglycan layer in Gram-negative microbes, which instead take up the counterstain (Wilhelm et al., 2015). *Mycobacterium* species are unable to be Gram stained and are instead referred to as acid-fast (method developed by Ziehl-Neelsen), due to the observation that once they are stained with carbolfuchsin they cannot be decolorized by acids, a property first noted by Paul Ehrlich (Titford, 2010).

The mycobacterial cell wall is comprised of complex glycans, lipids and glycolipids and can be divided into three parts. Unlike Gram-negative bacteria, mycobacteria have an asymmetric cytoplasmic membrane with an outer leaflet containing both phospholipids together with phosphatidyl-*myo*-inositol mannosides (PIMs, Figure 1C, first glycolipid), lipomannans (LMs, Figure 1C, second glycolipid), lipoarabinomannans (LAMs, Figure 1C, third glycolipid), and mannose-capped LAMs (ManLAMs, not shown); while the inner membrane is comprised mostly of phospholipids and PIMs. The central part of the mycobacterial cell wall is known as the mycolyl-arabinogalactan-peptidoglycan (mAGP) complex. The mAGP begins with a peptidoglycan layer that is also atypical consisting of GlcNAc alternating with MurNAc or MurNGlyc (N-glycolylmuramic acid created by hydroxylating the acetyl methyl group of MurNAc) (Rimal et al., 2022). Then, there is an extensive network of arabinogalactans which are anchored to the peptidoglycan through a GlcNAc-GlcNAc-rhamnose (Rha) linker to MurNAc *via* their reducing end galactose (Gal) residues residues (Harrison et al., 2016). The non-reducing end of the arabinogalactans are subsequently covalently attached through arabinose (Ara) to mycolic acids which form the inner leaflet of the outermost mycomembrane layer (Figure 1C). The outer leaflet of the mycomembrane contains additional mycolic acids modified with trehalose (trehalose mono- and di-mycolates), acylated trehaloses (di-, tri- and penta-), and trehalose sulfolipids (the latter are not shown). In addition, the outer leaflet contains phenolic glycolipids with methylated rhamnose and fucose residues linked through phenol to an unusual lipid known as phthiocerol dimycocerosate (PDIM) (Batt et al., 2020) (Figure 1C) and a capsular polysaccharide (not shown). For reviews on mycobacteria, see (Guerin et al., 2010; Abrahams and Besra, 2018; Garcia-Vilanova et al., 2019), and to examine the enzymes involved in the biosynthesis of these *M. tuberculosis* glycans, the reader should go to MicroGlycoDB (MicroGlycoDB, 2022).

## A model microbe, *Campylobacter jejuni*

**FIGURE 2:**
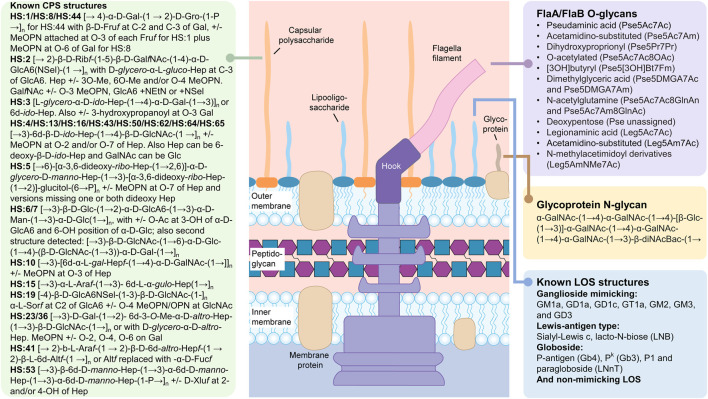
Carbohydrate structures synthesized by *Campylobacter jejuni*. The summarized structures include the capsular polysaccharides (CPS), the lipooligosaccharides (LOS) and the N- and O-linked glycoprotein glycans. N-glycans modify a large number of glycoproteins while O-glycans are found exclusively on the FlaA and FlaB subunits of the flagellum. Peptidoglycan sugars are the same as described in Figure 1. The free oligosaccharides (fOS) derived from the N-glycan pathway are not shown (see text). All sugars are in the pyranose form unless otherwise noted. Pse5Ac7Ac, pseudaminic acid (5,7-diacetamido-3,5,7,9-tetradeoxy-L-*glycero*-*α*-L-*manno* nonulosonic acid; Leg5Ac7Ac), legionaminic acid (5,7-diacetamido-3,5,7,9-tetradeoxy-D-*glycero*-*β*-D-*galacto*-nonulosonic acid); Fm, formyl; Gal, galactose; Gro, glycerol; Fru*f*, fructose in furanose configuration; MeOPN, methyl phosphoramidate; Rib, ribose; GalNAc, N-acetylgalactosamine; GlcA6, glucuronic acid; Hep, heptose; GlcNAc, N-acetylglucosamine; Glc, glucose; Man, mannose; Ara, arabinose; Alt, altrose; Sel, serinol; Xlu, xylulose. Structures were found in: (McNally et al., 2005; McNally et al., 2006; Hitchen et al., 2010; Houliston et al., 2011; Monteiro et al., 2018; Riegert et al., 2021).

*Campylobacter jejuni* is a human gastrointestinal pathogen that exists as a gut commensal in most animals and is found at particularly high densities in birds. For the last 2 years, the Centers for Disease Control have reported that this Gram-negative microbe is the leading cause of bacterial foodborne diarrheal disease in the United States (CDC, 2022). Source attribution studies in higher-income countries repeatedly demonstrate that contaminated poultry products are the primary source for infection, and as few as 500 bacterial cells have been reported to initiate infection (Kothary and Babu, 2001; Sheppard et al., 2009). *C. jejuni* is also a significant cause of bacterial diarrhea in low-and-middle income countries where infection leads to high rates of morbidity and mortality, particularly in infants, where the microbe has been associated with growth stunting and deficits in cognitive development (Amour et al., 2016). The majority of *C. jejuni* isolates express surface glycans mimicking human gangliosides (Figure 2), which play a key role in triggering Guillain-Barré Syndrome (GBS) through formation of anti-ganglioside antibodies that fix complement onto human neurons (Goodfellow and Willison, 2016). GBS is an acute inflammatory demyelinating neuropathy that is not only the leading cause of paralysis since the near-eradication of polio, but has been increasingly associated with outbreaks in multiple countries (Ramos et al., 2021).

Although campylobacters are well-recognized as ubiquitous pathogens infecting up to 85% of infants in low resource countries (Amour et al., 2016), they are arguably even more notorious in the glycoscience community for their ability to synthesize a diverse set of glycoconjugates (Figure 2). For example, *C. jejuni* was the first bacterium described to possess a pathway for N-linked protein glycosylation (Szymanski et al., 1999). *C. jejuni* also generates an array of nonulosonic acid derivatives that are O-linked to its flagella (Hitchen et al., 2010), and another 9-carbonsugar, sialic acid to its lipooligosaccharides to mimic host gangliosides, among other common structures... (Houliston et al., 2011). Furthermore, *C. jejuni* isolates synthesize capsular polysaccharides (CPS) differing in structure from strain to strain and representing 47 different serotypes to-date, in addition to exhibing strain-specific phase-variation (Guerry et al., 2012; Monteiro et al., 2018). For *C. jejuni*, each cell is heterogeneous within a population simply due to the existence of short homopolymeric runs of guanines or cytosines in multiple genes within the genome. These gene sequences are hypervariable due to the high rate of slipped-strand mispairing that occurs during replication as a result of an inefficient DNA repair system lacking key enzymes found in other microbes (Parkhill et al., 2000). The end result is that *C. jejuni* can typically survive most stresses instantaneously since there is a high probability that some cells exist within the population that are impervious to the stressor and not only survive, but thrive.

One phase-variable structure we originally identified in *C. jejuni* strain NCTC 11168 [the first genome-sequenced isolate of *C. jejuni* (Parkhill et al., 2000)] is the O-methyl phosphoramidate (MeOPN) that can be transfered to Gal*f*NAc or D-*glycero* L-*gluco*-heptose (Hep) residues of its CPS (Szymanski et al., 2003). The MeOPN modification, not previously described in nature, modifies at least 70% of *C. jejuni* CPS of varying serotypes (McNally et al., 2007). The unusual phosphorus-nitrogen bond is believed to predate the emergence of life and is created by the phosphorylation of glutamine through a *C. jejuni*-specific glutamine kinase (Taylor et al., 2017). Glutamine is also transfered by *C. jejuni* onto its O-linked pseudaminic acid residues, among many other unusual modifications (Hitchen et al., 2010). This is all done with a genome size approximately one-third that of *E. coli* and in an organism unable to catabolize most carbohydrates due to a lack of key enzymes from the Embden-Meyerhof-Parnas glycolysis pathway. Thus, with the exception of a catabolic fucose pathway we identified in some isolates (Stahl et al., 2011), *C. jejuni* strains are asaccharolytic and synthesize all their carbohydrates *de novo*. It is therefore not surprising that *C. jejuni* is a rich source of enzymes for glycobiologists to discover, but it is also difficult to imagine how this pathogen can thrive and outcompete other microbes that possess a greater capacity for nutrient acquisition and catabolism.

## *C. jejuni* variation from strain to strain

As with most bacteria, each strain within a species will express diverse carbohydrate structures and this diversity often leads to a serotyping scheme for that particular microbe. For *C. jejuni*, the CPS is the immunodominant structure contributing to the heat stable (HS) typing scheme originally developed by Penner and Hennessy (1980). Currently, researchers use molecular methods such as multilocus sequence typing (MLST) or whole genome sequencing to differentiate strains, but previously most researchers would distinguish strains based on their recognition by a panel of specific CPS antibodies that were used for typing. Unlike proteins, carbohydrates do not denature or alter their conformation when heated, and therefore the immunogenic CPS sugar epitopes are considered heat-stable. *C. jejuni* is divided into 47 different HS serotypes that in most cases correspond to very different CPS structures [Figure 2, and summarized by (Monteiro et al., 2018)]. However, not only does each *C. jejuni* strain express a different CPS structure, each strain also expresses a different collection of nonulosonic acid derivatives that are O-linked to its flagella, and a group of ganglioside (or other) mimics as part of its lipooligosaccharides. Figure 2 summarizes the diverse array of carbohydrate structures reported for *C. jejuni* to date and Supplementary Tables S1–S4 summarize the gene clusters and encoded enzymes responsible for the biosynthesis of these structures in *C. jejuni* 11168.

Although it would appear that with such diversity, it would be difficult for the microbe to be recognized by any bacteriophage, these viruses continue to evolve along with their hosts and often provide clues on structural aspects that are similar among strains of the same species. One such example is the MeOPN residue mentioned above that modifies most CPS structures (Figure 2) and, in comprehensive work done together with the Brondsted group, it was shown that many *C. jejuni* phages recognize this common residue on the bacterial CPS (Gencay et al., 2018). Similarly, it is known that *C. jejuni* strains cannot assemble their flagellar filaments without the O-linked glycans that can modify up to 19 different Ser/Thr sites per FlaA/FlaB subunit (Thibault et al., 2001). In strains capable of synthesizing both legionaminic acid (Leg) and pseudaminic acid (Pse), biosynthesis of Leg can be inactivated without an impact on flagellar biosynthesis, but Pse-derived monosaccharides are essential for filament assembly and motility. Interestingly, all *C. jejuni* phages characterized to-date possess a non-structural protein which we have named FlaGrab for its ability to specifically bind to flagellar filaments (Sacher et al., 2020). Through mutagenesis studies and screening on the Consortium for Functional Glycomics glycan array, it was determined that FlaGrab is specific for only acetamidino-modified Pse, a modification once again commonly found on the flagella of different *C. jejuni* isolates (Javed et al., 2015).

There is also one *C. jejuni* carbohydrate structure that remains relatively invariant, and that is the N-linked heptasaccharide attached to asparagine residues of at least 80 different glycoproteins (Figure 2) (Young et al., 2002; Cain et al., 2019) and also released as free oligosaccharide (fOS) into the periplasmic space of the microbe (Nothaft et al., 2009). Early studies demonstrated that fOS was actively released by the oligosaccharyltransferase, PglB, in response to osmotic stress (Nothaft et al., 2009); and that fOS concentrations were so abundant that the oligosaccharide could be readily purified from cell culture (Dwivedi et al., 2013) and detected within whole cells by a method known as high resolution magic angle spinning (HR-MAS) NMR (Szymanski et al., 2003). But, what propelled *C. jejuni* to star status in the microbial glycobiology field was that *C. jejuni* was the first bacterium shown to possess an N-glycosylation pathway, and the subsequent demonstrations that this pathway could be functionally transfered into *E. coli* to generate recombinant glycoproteins and novel glycoconjugate vaccines (Wacker et al., 2002; Harding et al., 2019; Harding and Feldman, 2019). Other key discoveries during this time were the observations that the *C. jejuni* oligosaccharyltransferase, PglB, attached sugars to a defined sequon (D/E-X_1_-N-X_2_-S/T where X_1_/X_2_ cannot be proline) and that PglB would transfer any oligosaccharide (even derived from an unrelated LPS or CPS pathway) provided that the sugars were assembled on the same lipid used by the *C. jejuni* pathway i.e., undecaprenylphosphate (Und-P, also known as bactoprenyl) and the reducing-end sugar was an N-acetylhexosamine (Feldman et al., 2005; Kowarik et al., 2006). Due to the commonality of this N-glycan structure among all *C. jejuni* strains and the requirement for N-glycan expression for *C. jejuni* survival in chickens, we have been exploiting this heptasaccharide in the development of glycoconjugate poultry vaccines for the prevention of human foodborne illness (Nothaft et al., 2016; Nothaft et al., 2017; Nothaft et al., 2021b). For more reading on N-linked protein glycosylation in bacteria or bacterial glycoprotein biosynthesis, see (Nothaft and Szymanski, 2019) and (Nothaft and Szymanski, 2022), respectively.

## *C. jejuni* variation within a single strain

**FIGURE 3:**
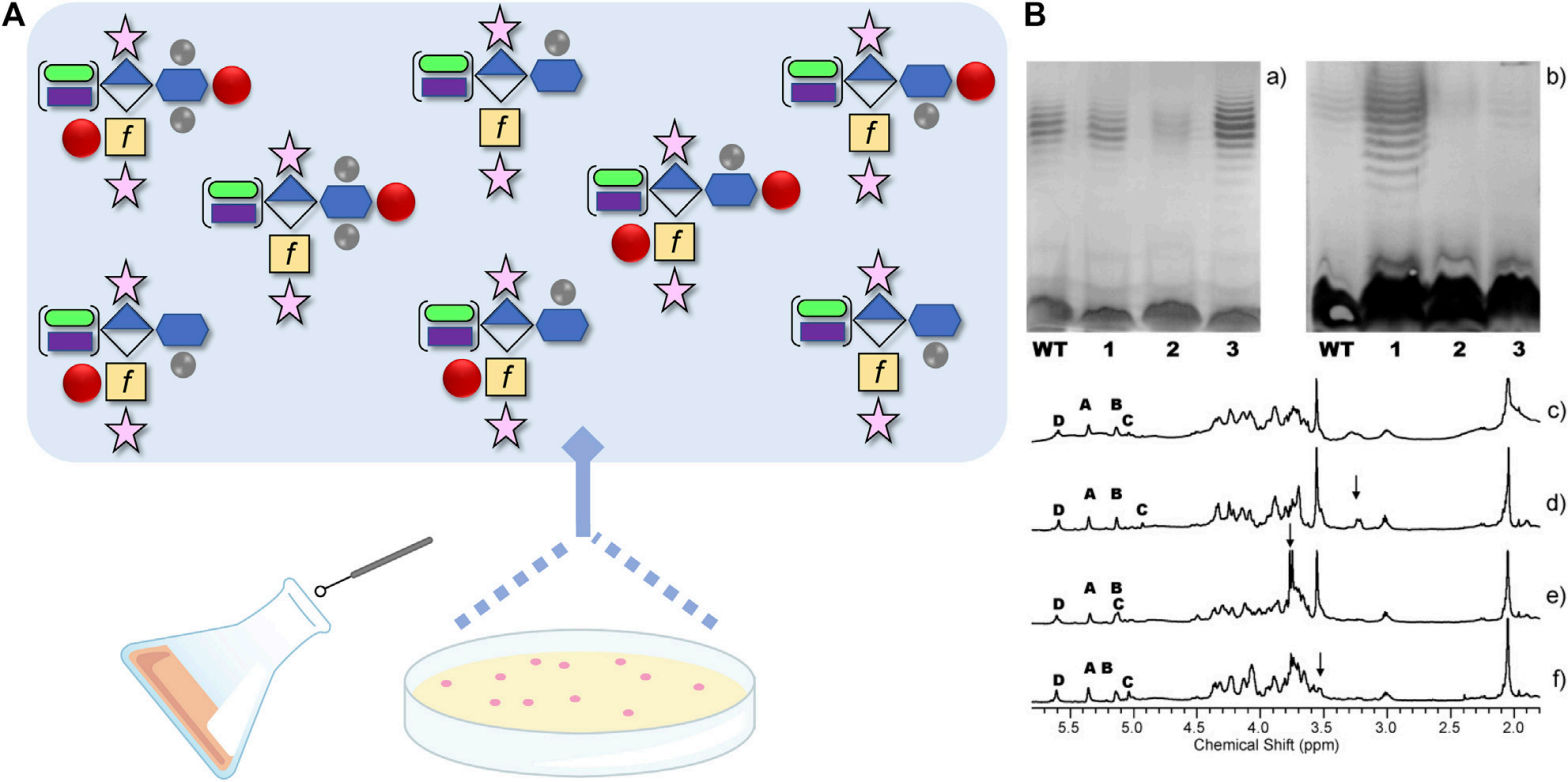
**(A)** Schematic demonstrating the reported capsular polysaccharide (CPS) structures originating from *Campylobacter jejuni* strain NCTC 11168, from ~ 1000 possible structures, using the Symbol Nomenclature for Glycans with some modifications. The CPS structure shown is the [→ 2)-β-D-Rib*f*-(1–5)-β-D-Gal*f*NAc-(1–4)-α-D-GlcA6(NSel)-(1 →]_n_ repeating unit with D-*glycero*-α-L-*gluco-*Hep (blue hexagon) at C-3 of GlcA (diamond). The Hep can be further modified+/-3O-Me, 6O-Me (methyl groups are grey circles) and/or O-4 MeOPN (methyl phosphoramidate drawn as red circle). In addition, the Gal*f*NAc (yellow square with *f* to designate furanose form) can be modified at O-3+/-MeOPN, and GlcA6 can be modified with either NSel (N-serinol, green oval) or NEtN (N-ethanolamine, purple rectangle). Ribose is shown as the pink star. **(B)** Original image from Szymanski et al., 2003 showing differences in CPS structures (c-f), and their corresponding silver-staining (a) and immunoreactivity (b) from single colony isolates originating from same culture. (a) Silver-stained deoxycholate-PAGE and (b) western blot detected with HS:2 typing sera both showing: lane 1, NCTC 11168 wildtype population; lane 2, 11168 variant one; lane 3, 11168 variant two; and lane 4, 11168 variant 3. (c-f) High resolution magic angle spinning (HR-MAS) NMR spectra of the wildtype population (c), variant 1 with arrow indicating ethanolamine resonance (d), variant 2 with arrow indicating MeOPN modification identified for the first time (e), and variant 3 with the arrow indicating loss of OMe resonance (f). Anomeric resonances in c-f are labeled A (Rib*f*), B (GlcA6), C (Gal*f*NAc), and D (Hep). See (Szymanski et al., 2003) for more information.

In *C. jejuni*, the predominant mechanism for variation of carbohydrate surface structures is through the random method of slipped-strand mispairing. First described by Parkhill et al. (2000) when comparing shotgun sequences of *C. jejuni* 11168, the authors observed variability in the homopolymeric tracts of primarily poly G:C runs in 23 different genes (with the potential for variability in nine others), and many of these genes were in clusters encoding enzymes involved in CPS, LOS and O-glycan biosynthesis. Several studies were subsequently published demonstrating the variability of these three glycoconjugate structures and the ability for *C. jejuni* to escape selective pressures by rapidly switching to a beneficial structure type, most likely because that structure type already existed amongst the population ((Guerry et al., 2002), see below). In fact, we first discovered the MeOPN by selecting individual colonies of *C. jejuni* 11168, restreaking each of those colonies onto a single agar plate, and then comparing their CPS structures by HR-MAS NMR, a method that allowed us to directly look at 40 μl of intact cells (Figure 3, (Szymanski et al., 2003)). Examination of these single colonies did indeed show differences in CPS structures, including the identification of one variant capable of expressing MeOPN at O-3 of Gal*f*NAc in the CPS (Szymanski et al., 2003). We subsequently identified two MeOPN transferases and showed that the second enzyme transfers MeOPN onto O-4 of D-*glycero*-L-*gluco*-Hep of CPS (McNally et al., 2007). These observations explained why MeOPN was not described in our original CPS structure publication (St Michael et al., 2002) - it was due to the fact that the wildtype 11168 strain used for structural analyses was comprised of a majority of cells with genes encoding both MeOPN transferases in the off-state. The single colony study, and later studies demonstrated that not only were the two MeOPN residues variable, but that the phosphoramidate could also exist with and without the Me group (McNally et al., 2007). In addition, we observed variable 3-OMe and 6-OMe groups attached to the same heptose, and glucuronic acid residues modified with either N-ethanolamine (Szymanski et al., 2003) or what was subsequently shown to be N-serinol (Riegert et al., 2021). Taken together, if all eight of these variable modifications could be generated randomly, this indicates that *C. jejuni* 11168 alone could express 2^10^ = 1,024 different CPS structures, and several of these structures have already been observed (Figure 3).

With access to so many possible CPS structures, it is now simpler to comprehend how *C. jejuni* 11168 is capable of adapting quickly to external stresses, but we wanted to determine whether this adaptation can be observed experimentally. To test this, we differentially infected two groups of chickens with *C. jejuni* (Holst Sorensen et al., 2012). In one group, we orally gavaged *C. jejuni* 11168 expressing MeOPN on its CPS and observed the same MeOPN-CPS on the isolates collected from the chicken cecal contents 6 days later, after the birds were euthanized. In the other group, we gavaged chickens with the same *C. jejuni* 11168 strain expressing MeOPN on its CPS, but also administered phage F336 previously shown to recognize the MeOPN moiety on *C. jejuni* CPS (Sorensen et al., 2011). Six days later, we examined the *C. jejuni* population that remained and discovered that all *C. jejuni* colonies that were isolated either showed loss of MeOPN expression or gained a 6-OMe group on the heptose that presumably obstructed MeOPN binding. It was remarkable that after just 6 days, all the *C. jejuni* cells colonizing the chickens were now phage resistant, and even more remarkable was the observation that both chicken groups showed similar quantities of *C. jejuni*, so the change in CPS structure did not impede growth or select for a strain less capable of colonizing chickens (Holst Sorensen et al., 2012).

Another example of *C. jejuni* 11168 rapid adaptation was observed when we were studying the ganglioside mimicking LOS (Patry et al., 2019). As mentioned above, many isolates of *C. jejuni* mimic human gangliosides (Figure 2), and it is this mimicry that is associated with inducing autoreactive antibodies involved in GBS development (Goodfellow and Willison, 2016). It is also known that cholera toxin, secreted by *Vibrio cholerae*, has nanomolar binding affinity for one particular ganglioside structure, GM1. These observations led us to question whether cholera toxin could also impact the growth of *C. jejuni* strains, such as 11168, capable of mimicking GM1. Co-incubation of 11168 with the toxin did indeed reduce growth of the strain and this affect was likely due to changes in membrane permeability due to LOS cross-linking by the pentameric binding subunits of cholera toxin (Patry et al., 2019). However, incubation of cholera toxin with *C. jejuni* 11168 selected for a population of cells lacking the GM1 mimic that had turned-off (through phase variation) the galactosyltransferase (CgtB, Supplementary Table S1) involved in adding the terminal Gal of the GM1 mimic (Patry et al., 2019).

## Common features for bacterial glycoconjugate biosynthesis

Although it has become increasingly apparent that microbes are capable of synthesizing a plethora of carbohydrate structures (and rapidly changing those structures too), there are some rules and/or commonalities that assist microbial glycobiologists in solving the complex relationships between glycoconjugate structure and enzymes involved in their biosynthesis that underpin the biological studies focused on understanding glycan function (Whitfield et al., 2022). To begin, carbohydrate synthesis and activation for transfer occurs in the cytoplasm and cytoplasmic membrane of all bacteria. As in eukaryotes, microbes typically use glycosyltransferases (Leloir enzymes) that recognize nucleotide sugars (e.g., UDP-Glc) as activated monosaccharides for glycosylation reactions. But microbes also possess non-Leloir enzymes that transfer sugars from lipids. These enzymes work together with PGT (phosphoglycosyltransferase) enzymes that are involved in adding the sugar to the lipid, which is usually Und-P.

As mentioned above, researchers are now capable of engineering novel glycoproteins with N-linked LPS and CPS based on the observation that LPS, and occasionally CPS, can be synthesized on Und-P (Feldman et al., 2005; Harding et al., 2019; Harding and Feldman, 2019). This lipid is used not only for the synthesis of the *C. jejuni* N-linked heptasaccharide, but it is also used for assembling peptidoglycan polymers of GlcNAc-MurNAc and for other sugar structures requiring pre-assembly prior to transport including O-linked oligosaccharides that are subsequently transferred *en bloc*, LPS O-antigen synthesis by all Gram-negative microbes, teichoic acid polymer assembly by all gram-positives, and specifically the *en bloc* Wzy-dependent pathway of CPS too.

Also, many polysaccharides include linker sugars at the reducing end that must be added prior to initiating assembly of the polysaccharide. This is seen in the *M. tuberculosis* arabinogalactans described above, but also in the synthesis of Gram-positive teichoic and lipoteichoic acids (Wenzel et al., 2020), as well as in S-layers (Schaffer and Messner, 2017), and some capsular polysaccharides (Willis and Whitfield, 2013). And once the building blocks are assembled on the cytoplasmic face of the inner membrane, they must be transported across the membrane by flippases in order to complete the assembly process. Alternatively, if the entire polysaccharide is assembled in the cytoplasm, then transport systems are engaged to move the polymer across the membranes to their final destination. Also, the signal for transport can range from adding a unique capping structure to a polysaccharide to initiate transport, and/or using a ruler type protein to “sense” completion of the synthesis (Whitfield et al., 2020).

Lastly, and of note in Figure 1, is that all glycoconjugate attachments and crosslinking in the cell wall, occurs through the use of MurNAc rather than GlcNAc. This is likely due to the fact that GlcNAc also serves other metabolic functions in the cell while MurNAc is strictly for use in peptidoglycan biosynthesis. This may also explain why bacteria such as *C. jejuni* and *Neisseria meningitidis* express lipooligosaccharides (LOS) rather than lipopolysaccharides (LPS). For the latter, the lipid A-core is synthesized in parallel with the O-antigen and then the two are combined to form LPS (Whitfield et al., 2022). However, O-antigens are assembled on Und-P, the same lipid used for *C. jejuni* N-glycan assembly (and for *N. meningitidis* O-glycan assembly too). It is possible that to avoid mixing up polysaccharides, the microbes have adapted to use Und-P only for protein glycosylation and synthesize LOS instead of LPS. Alternatively, in microbes such as *Pseudomonas aeruginosa* and *Acinetobacter baumannii,* the same carbohydrates that are assembled on Und-P are also added to proteins and to the LPS/CPS, respectively (Castric et al., 2001; Lees-Miller et al., 2013). So why does peptidoglycan assembly differ? The answer could lie in the fact that the pentapeptides are added to MurNAc-Und-P first, prior to GlcNAc addition and flipping across the membrane. It is clear that there is still much to learn from these fascinating systems, but bacteria need different methods to compartmentalize reactions when they lack the organelles that serve comparable functions in eukaryotes.

## Discussion

Trillions of microorganisms colonize our bodies and these inhabitants greatly impact both human health and disease. We now appreciate that these microbes do not exist in isolation, but rather are rapidly sensing and reacting to their surroundings. Previous analytical studies involved the isolation of one specific glycoconjugate structure meticulously purified from one microbe typically grown under nutrient rich conditions. Such a workflow allowed the researcher to take a snapshot of the most abundant form of that particular glycoconjugate produced by that specific microorganism, and these methodical experiments formed the framework for future glycomics studies. For example, the original CPS structure of *C. jejuni* 11168 that we published (St Michael et al., 2002), did not include the identification of the MeOPN modification that we subsequently showed played a significant role in bacteriophage recognition of the pathogen. However, without first determining the backbone structure, it would have been more difficult to make sense of the data obtained for the phase variants derived from single colony amplification. Furthermore, it then became simpler to determine enzyme function through a process of elimination by mutagenesis studies supplemented by HR-MAS NMR analyses to show that Cj1421 and Cj1422 were MeOPN transferases rather than glycosyltransferases as they were initially annotated (McNally et al., 2007).

It is important to emphasize that homology modeling of enzymes involved in carbohydrate biosynthesis and transfer typically gives clues into the type of reaction that is catalyzed, but rarely provides information on the monosaccharide that is involved. And since microbes are capable of synthesizing an endless repertoire of sugars, it is challenging to determine the function of enzymes until all the components of the glycoconjugate have been identified. For example, we did not know why so many enzymes were required for the synthesis of the *C. jejuni* CPS heptose until we discovered that the heptose configuration was unusual (D-*glycero*-L-*gluco*-Hep)—and the first to be described of its kind (St Michael et al., 2002). Similarly, at the time, GalNAc had not been described to exist in the furanose configuration in any other glycoconjugate, and the MeOPN structure had not previously been described in nature. Although it seems unusual to have identified three unique components in one *C. jejuni* CPS structure, one simply needs to look at the cell wall of *M. tuberculosis* to realize the limitless capacity microbes possess for synthesizing unusual glycoconjugates.

We also know that microbial surface carbohydrates do not remain fixed in time. Research, including our own, has demonstrated that *C. jejuni* randomly phase-varies the structures of its LOS, CPS and O-glycans, and also possesses the ability to turn on and off its CPS entirely. This indeed creates a moving target for any immune response or phage attack directed at these structures and helps to explain why a microbe with limited virulence factors has become a ubiquitous pathogen. Also, although not discussed here, other microbes are capable of regulating the expression of their surface carbohydrates in response to the combination of inputs they are receiving at any one time—and we cannot forget the influence of neighboring cells, whether microbial or host. Last year, Szymanski and Koropatkin (2021) were editors of a special edition entitled, Microbial transformation of the human glycobiome, which included a series of articles demonstrating how bacteria are capable of altering host glycoconjugate structures. Similarly, the host (and other microbes present in that particular niche) can alter bacterial carbohydrate structures both indirectly by creating selective pressures, but also directly by releasing enzymes that may cleave specific sugars, or lectins that bind to certain monosaccharides and thus prevent the microbe from adhering, or may target it for immune recognition.

The future of microbial glycobiology is endless! Even with a model microbe such as *C. jejuni* for which we have a comprehensive understanding of the glycan structures that the microbe is capable of synthesizing—and extensive knowledge about the enzymes involved in the biosynthesis of these structures [for *C. jejuni* 11168, see (MicroGlycoDB, 2022)], we still have very little understanding about which structures are actually expressed in the microbe’s natural environments and how they are impacted *in vivo*—and we know even less about other *C. jejuni* strains, or *Campylobacter* species (Bian et al., 2020). For example, we recently discovered that even the invariant *C. jejuni* N-glycan structure *can* be altered through phase-variation to synthesize a hexasaccharide lacking the glucose branch, and yet this variation is not regularly observed (Nothaft et al., 2021a). Interestingly, rather than a homopolymeric G-tract, the *pglI* gene encoding the glucosyltransferase, possessed a repetitive GAT stretch encoding aspartate residues responsible for the DxDD motif, found in 3-glycosyltransferase GT2 CAZy family members and needed for divalent cation coordination and nucleotide phosphodiester interaction of the donor (Brockhausen, 2014). In this case, functional PglI enzymes have a DDDDE sequence in the catalytic region, but variants with one less aspartate are non-functional (Nothaft et al., 2021a). More studies are needed to determine the impact of this alteration and why it is so infrequent.

Beyond *C. jejuni*, there is still so much to do! As mentioned above, researchers have historically investigated pathogenic microbes that were simple to grow to the large scales needed for glycoconjugate isolation and purification. And the microbes examined were typically model organisms commonly used among multiple labs, similar to *C. jejuni* 11168. Now scientists are appreciating the relevance of looking at more recent clinical isolates, comparing multiple isolates from a species, examining strains that are not normally pathogenic, and trying to culture the “unculturable” in order to obtain a broader understanding of their specific system—or to discover novel enzymes capable of being exploited in the bottomless microbial toolbox. As analytical instrumentation and methods continue to improve in sensitivity, researchers will also be capable of learning more about the fascinating field of microbial glycobiology.
