# Supplementary material for: Bacterial glycosylation, it’s complicated
Source: Front Mol Biosci. 2022 Sep 30;9:1015771. doi: 10.3389/fmolb.2022.1015771 (PMC9561416; doi:10.3389/fmolb.2022.1015771)
Supplement: Supplementary file 1 [file DataSheet1.docx]

***Supplementary Material***

Tables S1-S4 summarize the predicted or experimentally determined functions for enzymes encoded within the gene clusters known for the biosynthesis of *Campylobacter jejuni* NCTC 11168 lipooligosaccharide (LOS), capsular polysaccharide(CPS), N-linked and O-linked glycoproteins, along with additional reading materials. For further information, the reader should go to MicroGlycoDB (MicroGlycoDB, 2022).

Table S1. *Campylobacter jejuni* NCTC 11168 lipooligosaccharide (LOS) biosynthesis

| **Gene number** | **Gene name** | **Accession number**  **(UniProt)** | **Sugar/modification added** | **Function/special features** |
| --- | --- | --- | --- | --- |
| ***cj1132c*** |  | Q0P9C2 |  | Unknown, conserved hypothetical protein |
| ***cj1133*** | *waaC* | Q2TJD2 | heptose (Hep-I) | Heptosyltransferase I |
| ***cj1134*** | *htrB* | Q0P9C0 |  | Lipid A biosynthesis acyltransferase |
| ***cj1135*** |  | Q0P9B9 | β-1,4-glucose (Glc) to Hep-I and β-1,2-Glc to Hep-II | Two-domain glucosyltransferase:  β-1,4-glucosyltransferase (N- terminal domain) and β-1,2- glucosyltransferase (C-terminal  domain). In some *C. jejuni* strains, *cj1135* contains a frameshift mutation that disrupts the C-terminal domain, those strains are lacking Glc on HepII |
| ***cj1136*** |  | Q0P9B8 | β-1,3 galactose (Gal I) to HepII | β-1,3-galactosyltransferase |
| ***cj1137c*** |  | Q0P9B7 | α-1,2 Gal (Gal III) to GalI | α-1,2-galactosyltransferase |
| ***cj1138*** |  | Q0P9B6 | β-1,3 Gal (Gal II) to GalI | β-1,3-galactosyltransferase |
| ***cj1139c*** | *cgtB/wlaN* | Q0P9B5 | β-1,3 Gal | Terminal galactosyltransferase to β-1,4 N-acetylgalactosamine (GalNAc); neither mutant binds to cholera toxin (CT); phase variable genes |
| ***cj1140*** | *cstIII* | Q0P9B4 | α-2,3 N-acetyl neuraminic acid (Neu5Ac) | Transfers Neu5Ac (sialic acid) to GalII; *cgtA/neuA1* (*cj1143*), *neuB1* (*cj1141*) and *neuC1* (*cj1142*) are required for sialic acid biosynthesis |
| ***cj1141*** | *neuB1* | Q0P9B3 | Neu5Ac synthesis | Sialic acid synthase (N-acetylneuraminic acid synthetase) |
| ***cj1142*** | *neuC1* | Q0P9B2 | *N*-acetyl-mannosamine (ManNAc) synthesis | UDP-N-acetylglucosamine 2-epimerase / *N*-acetyl-mannosamine synthase |
| ***cj1143*** | *cgtA/neuA1* | Q0P9B1 | β-1,4 GalNAc to GalII and CMP-Neu5Ac synthesis | Transfers β-1,4-N-acetylgalactosamine to GalII; and CMP-Neu5Ac synthesis by the C-terminal domain of *cj1143* |
| ***cj1144c*** |  | Q0P9B0 |  | Unknown, phase variable |
| ***cj1146c*** | *waaV* | Q0P9A9 |  | Unknown, putative Glc transferase |
| ***cj1148*** | *waaF* | Q0P9A8 | heptose (Hep-II) | Heptosyltransferase II |
| ***cj1149c*** | *gmhA* | Q9PNE6 | D-*glycero*-α/β-D-*manno*-Hep for LOS/CPS | Sedoheptulose 7-phosphate isomerase (redundant with *cj1424c*) |
| ***cj1150c*** | *hldE* | Q6TG09 | D-*glycero*-D-*manno*-Hep-1,7-biphosphate and ADP-Hep transferase | Bifunctional: D-*glycero*-β-D-*manno*-heptose 7-phosphate kinase/D-*glycero*-β-D-*manno*-heptose 1-phosphate adenylyltransferase |
| ***cj1151c*** | *hldD* | Q0P9A5 | ADP-L-*glycero*-β-D-*manno*-Hep | ADP-*glycero*-manno-heptose 6-epimerase |
| ***cj1152c*** | *gmhB* | Q6TG07 | D-*glycero*-β-D-*manno*-Hep-1-P for LOS/CPS | D-*glycero*-D-*manno*-heptose 1,7-bisphosphate phosphatase |

Table S2. *Campylobacter jejuni* NCTC 11168 capsular polysaccharide (CPS) biosynthesis

| **Gene number** | **Gene name** | **Accession**  **number**  **(UniProt)** | **Sugar/modification added** | **Function/special features** |
| --- | --- | --- | --- | --- |
| ***cj1413c*** | *kpsS* | Q0P8K1 | Putative 3-deoxy-D-*manno*-oct-2-ulosonic acid (Kdo) transferase | Possibly adds first KDO from CMP-β-Kdo to putative lysophosphatidylglycerol forming CPS linker |
| ***cj1414c*** | *kpsC* | Q0P8K0 | Putative 3-deoxy-D-*manno*-oct-2-ulosonic acid (Kdo) transferase | Putative poly- β2,4- and/or β2,7 Kdo transferase forming CPS linker |
| ***cj1415c*** |  | Q0P8J9 | Nucleotide-activated (OPN) phosphoramidate biosynthesis | Cytidine diphosphoramidate kinase |
| ***cj1416c*** |  | Q0P8J8 | Nucleotide-activated OPN biosynthesis | Cytidine triphosphate (CTP):phosphoglutamine cytidylyltransferase |
| ***cj1417c*** |  | Q0P8J7 | Nucleotide-activated OPN biosynthesis | γ-glutamyl-CDP-amidate hydrolase |
| ***cj1418c*** |  | Q0P8J6 | Nucleotide-activated OPN biosynthesis | L-glutamine kinase |
| ***cj1419c*** |  | Q0P8J5 | Putative methyl (Me) transferase | OPN methyltransferase |
| ***cj1420c*** |  | Q0P8J4 | Putative Me transferase | OPN methyltransferase, phase variable |
| ***cj1421c*** |  | Q0P8J3 | OPN  transferase | Adds OPN to C3 of the β-D-Gal*f*NAc residue, phase variable |
| ***cj1422c*** |  | Q0P8J2 | OPN transferase | Adds OPN to C4 of D-*glycero*-α-L-*gluco*-heptose, phase variable |
| ***cj1423c*** | *hddC* | Q0P8J1 | GDP-D-*glycero*-α-D-*manno*-Hep | D-*glycero*-D-*manno*-heptose 1-phosphate guanosyltransferase |
| ***cj1424c*** | *gmhA2* | Q9PMN3 | D-*glycero*-α/β-D-*manno*-Hep  for LOS/CPS | D-sedoheptulose-7-phosphate isomerase (redundant with *cj1149c*) |
| ***cj1425c*** | *hddA* | Q0P8I9 | D-*glycero*-α-D-*manno*-Hep-1,7-PP | D-*glycero*-D-*manno*-heptose 7-phosphate kinase |
| ***cj1426c*** |  | Q0P8I8 | Me transferase, CPS modification | D-*glycero*-α-L-*gluco*-heptopyranose 6-O-methyltransferase, phase variable |
| ***cj1427c*** |  | Q0P8I7 | *cj1427,cj1430c, cj1428c* to GDP-D-*glycero*-α-L-*gluco*-Hep synthesis | Oxidation of GDP-D-*glycero*-α-D-*manno*-hep to GDP-D-*glycero*-4-keto-α-D-*lyxo*-hep |
| ***cj1428c*** |  | Q0P8I6 | *cj1427,cj1430c, cj1428c* to GDP-D-*glycero*-α-L-*gluco*-Hep synthesis | GDP-D-*glycero*-4-keto-β-L-*xylo*-hep reduction to GDP-D-*glycero*-β-L-*gluco*-heptose |
| ***cj1429c*** |  | Q0P8I5 | Unknown | Hypothetical protein, phase variable |
| ***cj1430c*** |  | Q0P8I4 | *cj1427,cj1430c, cj1428c* to GDP-D-*glycero*-α-L-*gluco*-Hep synthesis | GDP-D-*glycero*-4-keto-α-D-*lyxo*-hep 3,5-epimerization to to GDP-D-*glycero*-4-keto-β-L-*xylo*-hep |
| ***cj1431c*** | *hddC* | Q0P8I3 | GDP-Hep transferase to CPS | Capsular polysaccharide heptosyltransferase; (GDP-heptosyltransferase) |
| ***cj1432c*** |  | Q0P8I2 |  | Putative sugar transferase, mutant shows loss of CPS |
| ***cj1433c*** |  | Q0P8I1 | Unknown function | 9 repeats of the aa sequence KIDLNNT near the N-terminus belongs to radical SAM superfamily |
| ***cj1434c*** |  | Q0P8I0 |  | Putative sugar transferase,  mutant shows loss of CPS |
| ***cj1435c*** |  | Q0P8H9 | May remove P from serinol / Etn | Putative phosphatase |
| ***cj1436c*** |  | Q0P8H8 | Creates ethanolamine (Etn)-P | L-serine phosphate decarboxylation to ethanolamine phosphate |
| ***cj1437c*** |  | Q0P8H7 | Creates serinol (Sel)-P | Transamination of dihydroxyacetone phosphate to (S)-serinol phosphate, possibly phase variable |
| ***cj1438c*** |  | Q0P8H6 | Amide bond between GlcA and Sel-P or Etn-P | Amidoligase at C-terminus, GT2 transferase domain at N-terminus, mutant shows loss of CPS |
| ***cj1439c*** | *glf* | Q0P8H5 | Converts UDP-GalNAc to UDP-Gal*f*NAc | UDP-GalNAc mutase; also converts UDP-Gal to UDP-Gal*f in vitro* |
| ***cj1440c*** |  | Q0P8H4 |  | Putative sugar transferase,  mutant shows loss of CPS |
| ***cj1441c*** | *kfiD* | Q0P8H3 | GlcA biosynthesis | UDP-glucose 6-dehydrogenase |
| ***cj1442c*** |  | Q0P8H2 |  | Putative sugar transferase,  mutant shows loss of CPS |
| ***cj1443c*** | *kpsF* | Q0P8H1 |  | Capsule polysaccharide export system |
| ***cj1444c*** | *kpsD* | Q0P8H0 |  | Capsule polysaccharide export periplasmic protein |
| ***cj1445c*** | *kpsE* | Q0P8G9 |  | Capsule polysaccharide export inner membrane |
| ***cj1447c*** | *kpsT* | Q0P8G8 |  | Capsule polysaccharide export ATP-binding protein |
| ***cj1448c*** | *kpsM* | Q0P8G7 |  | Capsule polysaccharide export inner membrane |

Table S3. *Campylobacter jejuni* NCTC 11168 N-glycoprotein biosynthesis

| **Gene number** | **Gene name** | **Accession**  **number**  **(UniProt)** | **Sugar/modification added** | **Function/special features** |
| --- | --- | --- | --- | --- |
| ***cj1119c*** | *pglG* | Q0P9D5 | No known function in N-glycan biosynthesis | Putative integral membrane protein, unknown function |
| ***cj1120c*** | *pglF* | Q0P9D4 | diNAcBac biosynthesis | UDP-N-acetyl-α-D-glucosamine C6 dehydratase |
| ***cj1121c*** | *pglE* | Q0P9D3 | diNAcBac biosynthesis | UDP-4-keto-6-deoxy-GlcNAc C4 aminotransferase |
| ***cj1122c*** | *unknown* | Q0P9D2 | No function in N-glycan biosynthesis | putative integral membrane protein |
| ***cj1123c*** | *pglD* | Q0P9D1 | diNAcBac biosynthesis | UDP-N-acetylbacillosamine N-acetyltransferase |
| ***cj1124c*** | *pglC* | Q0P9D0 | UDP-diNAcBac transferase to form Und-PP-diNAcBac | Undecaprenyl phosphate N,N'-diacetylbacillosamine 1-phosphate transferase |
| ***cj1125c*** | *pglA* | Q0P9C9 | α-1,3 GalNAc | α-1,3-N-acetylgalactosaminyltransferase |
| ***cj1126c*** | *pglB* | Q0P9C8 | heptasaccharide transfer to Asn | oligosaccharide transferase (OTase) to N-glycosylate proteins and release fOS |
| ***cj1127c*** | *pglJ* | Q0P9C7 | α-1-4 GalNAc | α-1,4-N-acetylgalactosaminyltransferase |
| ***cj1128c*** | *pgl* | Q0P9C6 | β-1-3 Glc | β-1,3-glucosyltransferase |
| ***cj1129c*** | *pglH* | Q0P9C5 | 3 x α 1-4 GalNAc | α-1,4-N-acetyl-galactosaminyltransferase,  GalNAc-polymerase |
| ***cj1130c*** | *pglK* | Q0P9C4 | Flippase | Flips assembled LLOs from cytoplasm into the periplasm |
| ***cj1131c*** | *gne* | Q0P9C3 | UDP-GalNAc and UDP-Gal synthesis | UDP-Glc/GlcNAc 4-epimerase required for LOS, CPS and *N*-linked glycosylation |

Table S4. *Campylobacter jejuni* NCTC 11168 O-glycoprotein (FlaA/FlaB only) biosynthesis

| **Gene number** | **Gene name** | **Accession**  **number**  **(UniProt)** | **Function/special features** |
| --- | --- | --- | --- |
| ***cj1293*** | *pseB* | Q0P8W4 | UDP-GlcNAc-specific C4,6 dehydratase/C5 epimerase to create UDP-2-Ac-2,6-dideoxy-β-L-*arabino*-4-hexulose  First step in Pse5Ac7Ac biosynthesis |
| ***cj1294*** | *pseC* | Q0P8W3 | C4 aminotransferase creating UDP-4-amino-4,6-dideoxy-β-L-AltNAc  Second step in Pse5Ac7Ac biosynthesis |
| ***cj1295*** |  | Q0P8W2 | Dimethylglyceric acid biosynthesis or transferase, possibly phase variable |
| ***cj1296*** |  | Q0P8W1 | Hypothetical protein, possibly phase variable |
| ***cj1297*** |  | Q0P8W0 | Hypothetical protein, possibly phase variable |
| ***cj1298*** | *legH* | Q0P8V9 | Leg biosynthesis, eighth step, N-acetyltransferase |
| ***cj1299*** | *acpP2* | Q0P8V8 | Putative acyl carrier protein |
| ***cj1300*** |  | Q0P8V7 | Putative SAM domain containing methyltransferase |
| ***cj1301*** |  | Q0P8V6 | Hypothetical protein |
| ***cj1302*** |  | Q0P8V5 | Putative HAD-superfamily phosphatase, subfamily IIIC |
| ***cj1303*** | *fabH2* | Q9PMZ6 | Putative 3-oxoacyl-[acyl-carrier-protein] synthase |
| ***cj1304*** | *acpP3* | Q0P8V3 | Putative 3-oxoacyl-[acyl-carrier-protein] synthase |
| ***cj1305c*** |  | Q2M5Q3 | Hypothetical protein, phase variable |
| ***cj1306c*** |  | Q0P8V1 | Hypothetical protein, phase variable |
| ***cj1307*** |  | Q0P8V0 | Putative amino acid activating enzyme |
| ***cj1308*** | *acpP4* | Q0P8U9 | Putative acyl carrier protein |
| ***cj1309c*** |  | Q0P8U8 | Hypothetical protein |
| ***cj1310c*** |  | Q0P8U7 | Hypothetical protein, phase variable |
| ***cj1311*** | *pseF* | Q0P8U6 | Acylneuraminate cytidylyltransferase creating CMP-Pse5Ac7Ac  Sixth and final step in Pse5Ac7Ac biosynthesis |
| ***cj1312*** | *pseG* | Q0P8U5 | Nucleotidase creating 2,4-diNAc-2,4,6-trideoxy-β-L-Alt  Fourth step in Pse5Ac7Ac biosynthesis |
| ***cj1313*** | *pseH* | Q0P8U4 | N-acetyltransferase creating UDP-2,4-diNAc-2,4,6-trideoxy-β-L-Alt  Third step in Pse5Ac7Ac biosynthesis |
| ***cj1314c*** | *hisF* | Q0P8U3 | Imidazole glycerol phosphate synthase subunit |
| ***cj1315c*** | *hisH* | Q0P8U2 | Imidazole glycerol phosphate synthase subunit |
| ***cj1316c*** | *pseA* | Q0P8U1 | Pseudaminic acid acetamidino biosynthesis |
| ***cj1317*** | *pseI* | Q0P8U0 | Pse synthetase creating Pse5Ac7Ac  Fifth step in Pse5Ac7Ac biosynthesis |
| ***cj1318*** | *maf1* | Q0P8S3 | Motility accessory factor (function unknown), phase variable |
| ***cj1319*** | *legB* | Q0P8T8 | Leg biosynthesis, Sixth step NAD-dependent 4,6-dehydratase |
| ***cj1320*** | *legC* | Q0P8T7 | Leg biosynthesis, Seventh step PLP-dependent aminotransferase |
| ***cj1321*** |  | Q0P8T6 | Putative transferase, upstream phase variable |
| ***cj1322*** |  | Q0P8T5 | Hypothetical protein |
| ***cj1323*** |  | Q0P8T4 | Hypothetical protein |
| ***cj1324*** | *ptmG* | Q0P8T3 | Leg biosynthesis (loss of Leg5Am7Ac and Leg5AmNMe7Ac in mutant) – encodes CMP-Leg5Am7Ac acetamidino-synthase |
| ***cj1325*** | *ptmH* | Q0P8T2 | Leg biosynthesis – encodes CMP-Leg5Am7Ac acetamidino -N-methyltransferase, phase variable |
| ***cj1327*** | *legI* | Q0P8T1 | Leg biosynthesis, tenth step legionaminic acid synthase |
| ***cj1328*** | *legG* | Q0P8T0 | Leg biosynthesis, ninth step NDP-sugar hydrolase / 2-epimerase |
| ***cj1329*** | *ptmE* | Q0P8S9 | Leg biosynthesis, fourth step GlcN-1-P guanylyltransferase |
| ***cj1330*** | *ptmF* | Q0P8S8 | Leg biosynthesis, second step isomerase creating GlcN-6-P |
| ***cj1331*** | *legF* | Q0P8S7 | Leg biosynthesis, eleventh step CMP-legionaminic acid synthetase |
| ***cj1332*** | *ptmA* | Q0P8S6 | Leg biosynthesis, first step glutaminase |
| ***cj1333*** | *pseD* | Q0P8S5 | Pse5Ac7Am transferase |
| ***cj1334*** | *maf3* | Q0P8S4 | Motility accessory factor (function unknown) |
| ***cj1335*** | *maf4* | Q0P8S3 | Motility accessory factor (function unknown), phase variable |
| ***cj1336*** | *pseE* | Q0P8S2 | Pse5Ac7Ac transferase |
| ***cj1338c*** | *flaB* | P56964 | Flagellin subunit B |
| ***cj1339c*** | *flaA* | P56963 | Flagellin subunit A |
| ***cj1340*** |  | Q0P8R9 | Conserved hypothetical protein |
| ***cj1341*** | *maf6* | Q0P8R8 | Motility accessory factor (function unknown) |
| ***cj1342*** | *maf7* | Q0P8R7 | Motility accessory factor (function unknown), phase variable |
| ***cj1407c*** | *pgmL* | Q0P8K7 | Leg biosynthesis, third step phosphoglucosamine mutase |
| ***cj0821*** | *glmU* | Q0PA69 | Leg biosynthesis, fifth step N-acetyltransferase |

**References**

Gilbert, M., Karwaski, M.F., Bernatchez, S., Young, N.M., Taboada, E., Michniewicz, J., et al. (2002). The genetic bases for the variation in the lipo-oligosaccharide of the mucosal pathogen, *Campylobacter jejuni.* Biosynthesis of sialylated ganglioside mimics in the core oligosaccharide. *J Biol Chem* 277(1)**,** 327-337. doi: 10.1074/jbc.M108452200.

Howard, S.L., Jagannathan, A., Soo, E.C., Hui, J.P., Aubry, A.J., Ahmed, I., et al. (2009). *Campylobacter jejuni* glycosylation island important in cell charge, legionaminic acid biosynthesis, and colonization of chickens. *Infect Immun* 77(6)**,** 2544-2556. doi: 10.1128/IAI.01425-08.

Huddleston, J.P., Anderson, T.K., Girardi, N.M., Thoden, J.B., Taylor, Z., Holden, H.M., et al. (2021). Biosynthesis of D-*glycero*-L-*gluco*-Heptose in the capsular polysaccharides of *Campylobacter jejuni*. *Biochemistry* 60(19)**,** 1552-1563. doi: 10.1021/acs.biochem.1c00183.

Huddleston, J.P., Anderson, T.K., Spencer, K.D., Thoden, J.B., Raushel, F.M., and Holden, H.M. (2020). Structural Analysis of Cj1427, an Essential NAD-Dependent Dehydrogenase for the Biosynthesis of the Heptose Residues in the Capsular Polysaccharides of *Campylobacter jejuni*. *Biochemistry* 59(13)**,** 1314-1327. doi: 10.1021/acs.biochem.0c00096.

Huddleston, J.P., and Raushel, F.M. (2020). Functional Characterization of Cj1427, a Unique Ping-Pong Dehydrogenase Responsible for the Oxidation of GDP-D-*glycero*-α-D-*manno*-heptose in *Campylobacter jejuni*. *Biochemistry* 59(13)**,** 1328-1337. doi: 10.1021/acs.biochem.0c00097.

Karlyshev, A.V., Champion, O.L., Churcher, C., Brisson, J.R., Jarrell, H.C., Gilbert, M., et al. (2005). Analysis of *Campylobacter jejuni* capsular loci reveals multiple mechanisms for the generation of structural diversity and the ability to form complex heptoses. *Mol Microbiol* 55(1)**,** 90-103. doi: 10.1111/j.1365-2958.2004.04374.x.

McCallum, M., Shaw, G.S., and Creuzenet, C. (2013). Comparison of predicted epimerases and reductases of the *Campylobacter jejuni* D-altro- and L-gluco-heptose synthesis pathways. *J Biol Chem* 288(27)**,** 19569-19580. doi: 10.1074/jbc.M113.468066.

MicroGlycoDB (2022). [*https://microglycodb.test.glycosmos.org/*](https://microglycodb.test.glycosmos.org/) [Online]. [Accessed August 09 2022].

Parkhill, J., Wren, B.W., Mungall, K., Ketley, J.M., Churcher, C., Basham, D., et al. (2000). The genome sequence of the food-borne pathogen *Campylobacter jejuni* reveals hypervariable sequences. *Nature* 403(6770)**,** 665-668.

Poulin, M.B., Nothaft, H., Hug, I., Feldman, M.F., Szymanski, C.M., and Lowary, T.L. (2010). Characterization of a bifunctional pyranose-furanose mutase from *Campylobacter jejuni* 11168. *J Biol Chem* 285(1)**,** 493-501. doi: 10.1074/jbc.M109.072157.

Rangarajan, E.S., Proteau, A., Cui, Q., Logan, S.M., Potetinova, Z., Whitfield, D., et al. (2009). Structural and functional analysis of *Campylobacter jejuni* PseG: a UDP-sugar hydrolase from the pseudaminic acid biosynthetic pathway. *J Biol Chem* 284(31)**,** 20989-21000. doi: 10.1074/jbc.M109.012351.

Riegert, A.S., Narindoshvili, T., Coricello, A., Richards, N.G.J., and Raushel, F.M. (2021). Functional Characterization of Two PLP-Dependent Enzymes Involved in Capsular Polysaccharide Biosynthesis from *Campylobacter jejuni*. *Biochemistry* 60(37)**,** 2836-2843. doi: 10.1021/acs.biochem.1c00439.

Riegert, A.S., Narindoshvili, T., and Raushel, F.M. (2022). Discovery and Functional Characterization of a Clandestine ATP-Dependent Amidoligase in the Biosynthesis of the Capsular Polysaccharide from *Campylobacter jejuni*. *Biochemistry* 61(2)**,** 117-124. doi: 10.1021/acs.biochem.1c00707.

Riegert, A.S., and Raushel, F.M. (2021). Functional and Structural Characterization of the UDP-Glucose Dehydrogenase Involved in Capsular Polysaccharide Biosynthesis from *Campylobacter jejuni*. *Biochemistry* 60(9)**,** 725-734. doi: 10.1021/acs.biochem.0c00953.

Schoenhofen, I.C., Vinogradov, E., Whitfield, D.M., Brisson, J.R., and Logan, S.M. (2009). The CMP-legionaminic acid pathway in Campylobacter: biosynthesis involving novel GDP-linked precursors. *Glycobiology* 19(7)**,** 715-725. doi: 10.1093/glycob/cwp039.

Sternberg, M.J., Tamaddoni-Nezhad, A., Lesk, V.I., Kay, E., Hitchen, P.G., Cootes, A., et al. (2013). Gene function hypotheses for the *Campylobacter jejuni* glycome generated by a logic-based approach. *J Mol Biol* 425(1)**,** 186-197. doi: 10.1016/j.jmb.2012.10.014.

Taylor, Z.W., Brown, H.A., Holden, H.M., and Raushel, F.M. (2017a). Biosynthesis of Nucleoside Diphosphoramidates in *Campylobacter jejuni*. *Biochemistry* 56(46)**,** 6079-6082. doi: 10.1021/acs.biochem.7b00905.

Taylor, Z.W., Brown, H.A., Narindoshvili, T., Wenzel, C.Q., Szymanski, C.M., Holden, H.M., et al. (2017b). Discovery of a Glutamine Kinase Required for the Biosynthesis of the O-Methyl Phosphoramidate Modifications Found in the Capsular Polysaccharides of *Campylobacter jejuni*. *J Am Chem Soc* 139(28)**,** 9463-9466. doi: 10.1021/jacs.7b04824.

Taylor, Z.W., and Raushel, F.M. (2018). Cytidine Diphosphoramidate Kinase: An Enzyme Required for the Biosynthesis of the O-Methyl Phosphoramidate Modification in the Capsular Polysaccharides of *Campylobacter jejuni*. *Biochemistry* 57(15)**,** 2238-2244. doi: 10.1021/acs.biochem.8b00279.

Thibault, P., Logan, S.M., Kelly, J.F., Brisson, J.R., Ewing, C.P., Trust, T.J., et al. (2001). Identification of the carbohydrate moieties and glycosylation motifs in *Campylobacter jejuni* flagellin. *J Biol Chem* 276(37)**,** 34862-34870. doi: 10.1074/jbc.M104529200.

Willis, L.M., and Whitfield, C. (2013). KpsC and KpsS are retaining 3-deoxy-D-manno-oct-2-ulosonic acid (Kdo) transferases involved in synthesis of bacterial capsules. *Proc Natl Acad Sci U S A* 110(51)**,** 20753-20758. doi: 10.1073/pnas.1312637110.
